# Effect of pH Buffer and Carbon Metabolism on the Yield and Mechanical Properties of Bacterial Cellulose Produced by *Komagataeibacter hansenii* ATCC 53582

**DOI:** 10.4014/jmb.2010.10054

**Published:** 2020-12-16

**Authors:** Zhaofeng Li, Si-Qian Chen, Xiao Cao, Lin Li, Jie Zhu, Hongpeng Yu

**Affiliations:** 1 School of Chemical Engineering and Light Industry, Guangdong University of Technology, Guangzhou 510006, P.R. China; 2 School of Chemical Engineering and Energy Technology, Dongguan University of Technology, Dongguan 523808, P.R. China; 3 Key Laboratory of Healthy Food Development and Nutrition Regulation of China National Light Industry, Dongguan University of Technology, Dongguan 523808, P.R. China; 4 Institute of Science and Technology Innovation, Dongguan University of Technology, Dongguan 523808, P.R. China

**Keywords:** Bacterial cellulose, pH buffer, mechanics, gluconic acid, glycerol, rheology

## Abstract

Bacterial cellulose (BC) is widely used in the food industry for products such as nata de coco. The mechanical properties of BC hydrogels, including stiffness and viscoelasticity, are determined by the hydrated fibril network. Generally, *Komagataeibacter* bacteria produce gluconic acids in a glucose medium, which may affect the pH, structure and mechanical properties of BC. In this work, the effect of pH buffer on the yields of *Komagataeibacter hansenii* strain ATCC 53582 was studied. The bacterium in a phosphate and phthalate buffer with low ionic strength produced a good BC yield (5.16 and 4.63 g/l respectively), but there was a substantial reduction in pH due to the accumulation of gluconic acid. However, the addition of gluconic acid enhanced the polymer density and mechanical properties of BC hydrogels. The effect was similar to that of the bacteria using glycerol in another carbon metabolism circuit, which provided good pH stability and a higher conversion rate of carbon. This study may broaden the understanding of how carbon sources affect BC biosynthesis.

## Introduction

Bacterial cellulose (BC) is a naturally occurring nanomaterial produced by some bacteria, such as those from the genus *Komagataeibacter*, consisting of a network of cellulose nanofibers connected by *β*-1-4-glycosidic bonds [1, 2]. Compared to other genera, *Komagataeibacter* is usually the genus of choice for research and food applications, due to its higher BC yield and purity [3, 4]. These attributes allow it good liquid absorption capacity, nontoxicity and good mechanical properties [5]. BC has long been used as a raw material for nata de coco, an indigenous coconut gel product used in bubble tea and other desserts in the Philippines and China [6]. In addition, BC may serve as a food ingredient to improve the texture and mouth feel of foods as an emulsifier and stabilizer for foods such as diary-based drinks and ice cream, respectively [7].

Despite the popularity of BC-based desserts, the cost of production has remained high, being limited by the carbon conversion rate and having to maintain mechanical properties optimized for consumer preference [8-11]. Generally, the yield of BC is affected by bacterial strain, medium composition and culture conditions (*e.g.*, pH, agitation rate, oxygen availability) [12-15]. Additionally, high-yielding bacterial strains also contribute to a more homogenous BC fibril network structure and corresponding mechanical properties (*i.e.*, extensibility, stiffness, viscoelasticity and poroelasticity) [14, 16, 17]. Previous reports have shown *Komagataeibacter hansenii* ATCC 53582 contains two additional operons involved in manipulating the Acs operons in cellulose synthesis [8, 18], potentially producing a higher yield of BC [17, 19, 20].

Regulating the bacterial carbon metabolism pathway during cellulose synthesis is an additional avenue to achieve higher BC-hydrogel yields while maintaining comparable mechanical properties. Metabolism of glucose as a carbon substrate is among a range of biochemical pathways including fructose, mannose, and ethanol-production as carbon can be effectively consumed by most *Komagataeibacter* bacteria [21-24]. However, gluconic acid and acetic acid are considered as undesired byproducts of cellulose synthesis from glucose and ethanol, respectively, and result in lower medium pH and production yield [25, 26]. It is well established that pH 4-7 is the optimal range for BC culture [11, 27]. To stabilize the pH, an effective buffer is essential for BC production to prevent the accumulation of acidic compounds in the medium that may have a negative effect on the structure of BC hydrogels [28, 29]. An alternative method is to choose carbon sources that the bacteria use only to synthesize cellulose rather than the acid by-products. Considering the pathways of carbon metabolism in *Komagataeibacter xylinus*, glycerol may be a good potential carbon source that may not significantly alter the pH [30].

In this study, the efficacy of BC production using *K. hansenii* ATCC 53582 with various buffer pH levels and at different starting pH values was investigated. The role of gluconic acid in cellulose synthesis was also evaluated using glucose, gluconic acid and glycerol as single or combined carbon sources. In addition, the effect of different carbon sources on the structure and mechanical properties of BC was also discussed.

## Materials and Methods

### BC Production Procedure

Bacterial strain *K. hansenii* ATCC 53582 was obtained from American Type Culture Collection (ATCC), USA. The inoculation process followed a previously described method with the following modification [31]: the bacteria were activated on Hestrin-Schramm (HS) agar plates (2 wt% glucose, 0.5 wt% peptone, 0.5 wt% yeast extract, 0.27 wt% Na_2_HPO_4_ and 0.115 wt% citric acid) [32] and were cultivated at 30°C, pH 5.0 for 3 days. In this study, the starting pH was adjusted by adding 1 N HCl or NaOH solution unless otherwise stated. The primary inoculation was carried out by transferring a single colony into the HS broth and statically cultivating it for 3 days at 30°C with a starting pH at 5. The harvested primary inoculation was vibrated to release the attached cells on the cellulose membranes. The bacteria were then scaled-up at 30°C with a 10% (v/v) inoculation. When the cultivation was completed, the BC pellicles were rinsed with deionized water for several times to remove the residual medium. Subsequently, the BC pellicles were transferred to a beaker containing 0.1 N NaOH, and then stirred and boiled at 95°C for 20 min. BC pellicles were then repeatedly washed with deionized water until the pH reached neutral, detected by using a pH indicator. The washed BC was either stored in 0.1 wt% potassium sorbate solution or pre-frozen in a refrigerator at -80°C for 6 h and then freeze-dried at -75°C for 48 h for further tests. The BC yield was calculated as freeze-dried BC weight per liter of the culture medium. The carbon conversion rates were defined as the weight of dried BC divided by the dry weight of the added carbon sources in the original medium.

### Buffers and Starting Culture pH

To investigate the effect of buffer and starting culture pH on the yields and mechanics of BC, all media used 2 wt% glucose, 0.5 wt% peptone, and 0.5 wt% yeast extract as the carbon and nitrogen source, and the composition of the buffer is shown in Table 1 [33, 34]. The starting pH gradients were set at 4.60, 5.00, 5.40, and 5.80 respectively. The control group was prepared by removing the phosphate and citrate in the original recipe of HS medium.

The concentration of glucose and gluconic acid in the culture medium was detected by using a Glucose Assay Kit and D-Gluconate/D-Glucono-d-lactone Assay Kit (both from Megazyme, Ireland) respectively. Following the manufacturer’s protocol, samples were mixed with deionized water at the ratio of 1:9 (v/v). The attached bacterial cells were removed by centrifuging at 10,000 ×*g* for 10 min. The absorbance of the specimens was measured by using an ultraviolet (UV) spectrophotometer (Evolution 200, Thermo Fisher, USA) to calculate the concentration of glucose or gluconic acid in the scale-up inoculation media.

### Gluconic Acid and Glycerol as Carbon Sources

Gluconic acid was added to the HS medium as additional carbon source and/or pH adjuster. In the medium supplemented with gluconic acid, the 1N HCl was no longer used to adjust the pH. Glycerol was used to replace glucose at the same concentration (2 wt%) in HS medium with other conditions maintained. HS medium was set as the control group.

### Scanning Electron Microscopy (SEM)

The freeze-dried BC pellicles were cut vertically from the top surface into small pieces of ca. 2 × 2 mm by using a sharp blade. Samples were mounted and gold-coated, and examined using scanning electron microscope (EM-30 Plus, COXEM, Korea) imaging under the following conditions: acceleration voltage at 5 kV and a working distance of 10 mm. All images were taken perpendicularly towards the top surface of the sample. Images were randomly taken from at least three different positions of three individual samples, with a series of magnifications increased from ×5,000, ×7,000, ×10,000 to ×20,000.

### X-Ray Diffraction (XRD)

XRD measurements of freeze-dried BC pellicles were performed on a Bruker diffractometer (UltimalV, Japan) running at 40 kV, 40 mA, CuK*α* radiation monochromated with a graphite sample monochromator. The diffractogram was recorded between 2*θ* angles of 10° to 30°.

Peak fitting was performed in Origin software (OriginLab, USA). Gauss function was used to fit the diffraction peaks obtained. For the fitting process, diffraction patterns were considered to be caused by the represented reflection of the 100, 010 and 110 crystal planes of the cellulose Iα allotrope, corresponding to 14.4°, 16.8°, and 22.6° of 2*θ* respectively, as well as the amorphous area centered at approximately 18.5° [35]. The crystallinity index (CI) was determined by the following equation:

$$\mathrm{CI}=\Sigma_{C}/\Sigma_{T}\times 100$$

Where Σ_C_ and Σ_T_ are the sum of areas under three crystalline peaks, and sum of areas under all diffraction peaks, respectively.

The dimension of the crystal was evaluated by using Scherrer’s expression [36].

D=Kλ/βcosθ

where *D* is the average crystalline width of a specific phase; *K* is a constant that varies with the method used to measure the breadth (K = 0.9); *λ* is the wavelength of incident X-rays (*λ* = 0.154 nm); *θ* is the center angle of the peak; *β* is the full width at half maximum (FWHM) of the reflection peak.

### Compression-Relaxation/Small Amplitude Oscillation (SAOS) Cycle Test

The mechanical and rheological properties of BC hydrogels were measured by using a rheometer (MCR 702 Rheometer, Austria) at a constant temperature of 25°C. Parallel plates with upper and bottom diameters of 40 mm and 60 mm were used. The upper and bottom plates were both pasted with fine emery paper (P240/S85, 58 μm roughness) to avoid slipping of the BC hydrogels. The BC hydrogel was placed in the center of the parallel plates. The initial gap (the distance between the upper plate and the bottom plate) was adjusted to the same height as the sample. The normal force was measured by a sensor (50 N).

The stiffness and recovery ability of the BC gels were investigated in the axial compression/relaxation test. The viscoelasticity was measured in the small amplitude oscillation (SAOS) test. The method followed the description provided elsewhere with slight modification [37, 38]. During axial compression, the BC gels were compressed by 100 μm (t_0_) at a constant speed (1 μm/s). After each compression step, the SAOS test was performed at a frequency of 10 rad/s and at a low constant shearing strain of 0.01% (chosen from the linear viscoelastic region) for 120 s. The storage and loss modulus (G′ and G′′) were recorded. A sequence of compression–relaxation/SAOS tests were carried out until the normal stress reached the limit of the sensor. The BC hydrogels were compressed from the initial thickness to the narrowest possible gap (typically 500–1,000 μm). At least two replicates were measured until a high degree of reproducibility was achieved.

## Results and Discussion

### Effect of Different Buffer and Starting pH on BC Production

As expected, a starting pH of 5 was the most suitable for BC production. The BC yields of *K. hansenii* ATCC 53582 in HS medium (phosphate buffer, ionic strength = 90 mM) was 4.63 g/l after 9 days of cultivation (Table 2), which was close to the result of some previous reports [39, 40]. However, during this period, the pH dropped from 5.0 to 3.5, which indicated that the phosphate buffer failed to neutralize the excess hydrogen ions produced by the bacteria during fermentation. The increasing hydrogen ions were possibly from the acidic by-products of the oxidized glucose [25, 26, 41]. To stabilize the pH, the ionic strength was enhanced from 90 mM to 600 mM in the original HS medium. In addition, a various buffers including the acetate, the phthalate and the citrate buffer at different ionic strength levels were also compared (Table 2). Compared with the targeting pH (4.60, 5.00, 5.40, and 5.80), the actual starting culture pH of all samples changed slightly in an allowable range due to the autoclave or other processes during the preparation. When the ionic strength of the phosphate buffer was raised from 90 mM to 600 mM, the bacteria lost their cellulose synthesis ability regardless of the starting pH levels. Additionally, in the modified HS medium that contained the acetate (ionic strength = 600 mM) or the citrate buffer (ionic strength = 200-250 mM), the synthesis of BC was also restrained. Only a small amount of translucent and incomplete cellulosic floccules appeared (Fig. S1 in supplementary materials). On the other hand, the bacterium produced 3.75-4.27 g/l of BC in the phthalate buffer which had a lower ionic strength (ca. 60-90 mM). However, if no buffer was used, when the starting pH was 5, the yields of BC in the control group (4.27 g/l) were significantly lower than the low ionic strength phosphate (BC yield = 4.63 g/l) or phthalate buffer (BC yield = 5.06 g/l).

The result indicated that the type and ionic strength of the buffer solution were both crucial for the bacteria to grow and produce cellulose. This finding was consistent with a previous report showing that the BC yield of strain *Gluconacetobacter xylinus* BCRC 12334 decreased in high ionic strength (300 mM) buffer [28]. However, this report also showed the acetate buffer promoted the BC yields to about 200% at 200 mM of ionic strength compared with phosphate buffer and successfully maintained the initial pH, which indicates that different *Komagataeibacter* strains may have different adaptabilities for specific buffer.

### Change of pH and BC Yield in Acetate/Phthalate Buffer and Gluconic Acid/Glycerol as Carbon Source Media

**Fig. 1:**
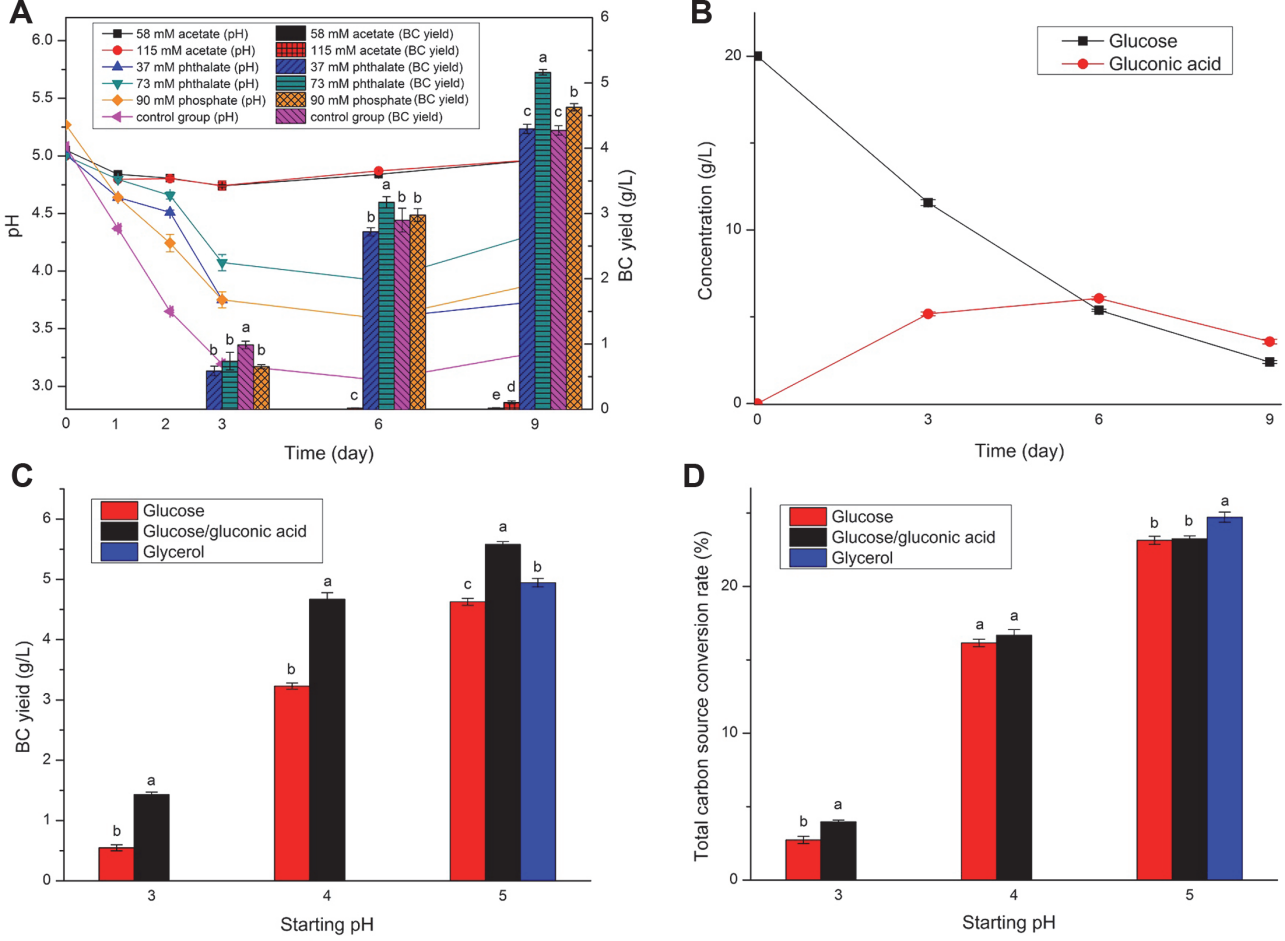
(A) Change of pH and BC yield in different buffer medium, (B) change of concentration of glucose/gluconic acid in the control group, (C) BC yields and (D) total carbon conversion rate in different carbon sources media with different starting pH.

The dynamic change of pH and BC yields in either acetate or phthalate buffer with different ionic strengths were shown in Fig. 1A. The bacterium only synthesized a small amount of cellulose in the acetate buffer even though the ionic strength was set to 50 mM. This indicates that the bacterium cannot effectively adapt to the acetate buffer like some other cellulose-producing strains [28, 42]. In contrast, the bacterium produced complete BC pellicles in the phthalate buffer media (Fig. S2 in supplementary materials), and the yields were 4.30 g/l and 5.16 g/l for the ionic strengths of 37 and 73 mM respectively. The bacterium maintained a high cellulose-producing ability in the 9 days of culture, showing prolonged behavior compared with some ‘normal yield’ strains whose peak synthesizing periods were normally 2-5 days [30, 43]. This long-term cellulose synthesis ability was similar with the high-BC-yield engineering strain that contained sucrose synthase genes in a previous report [44].

Although the bacteria produced more BC in the phthalate and phosphate buffer media than in the control group (Fig. 1A), the pH of both media dropped dramatically at the beginning stage of culture (0-3 days). At this stage, *Komagataeibacter* produced a large amount of gluconic acid while producing BC according to previous reports [28, 30]. In the control group, about 42% of the added glucose in the medium was quickly consumed, and ca. 61% of this consumed glucose was converted into gluconic acid in the first 3 days, which contributed to a large drop in pH (Fig. 1A). The pH dropped to a relative plateau at 3-6 days and the concentration of gluconic acid only increased by 0.91 g/l (Fig. 1B). At the later stage of the culture (6-9 days), the bacteria consumed ca. 39% of the synthesized gluconic acid to maintain the growth while most of the glucose (88%) had already been utilized. These distinct three stages of culture indicate the gluconic acid may not hinder BC production as much as expected, and the acidic environment may be important for the bacteria to grow when glucose is used as the carbon source. A previous report showed the bacteria can use gluconic acid as the sole carbon source for synthesis BC [45]. Therefore, gluconic acid was added to HS medium for use as an additional carbon source and pH adjuster. The initial pH was set to 3 to study whether the low starting pH condition was beneficial for BC production. The BC yields of *K. hansenii* ATCC 53582 in the glucose/gluconic acid media were shown in Fig. 1C. The result shows that the added gluconic acid (concentration = 4 g/l) significantly promoted the BC yield compared with using glucose as the sole carbon source (Fig. 1C), indicating that this substance may be a critical intermediate metabolite in the process of bacteria using glucose to synthesize cellulose. This was partially consistent with a previous report showing that glucuronic acid-based oligosaccharides can enhance BC production [39]. However, the synthesis ability of BC was still restrained at low starting pH levels (3-4) and the highest BC yield was achieved when the starting pH was 5, in which case the value was close to the optimal pH for glucose oxidase [46]. Since we expected to create an acidic environment similar to the peak period (3-6 days of cultivation) of cellulose synthesis by adding gluconic acids, this failure indicated that the lag phase of the bacteria growth may be more sophisticated. At this stage, the bacteria may selectively use the glucose and added gluconic acid to balance the pH of the medium, the growth of bacteria, and the synthesis of cellulose. The bacteria probably preferred to oxidize the present glucose to gluconic acid instead of directly using the added gluconic acid. Nevertheless, the conversion of glucose to gluconic acid was unavoidable. Moreover, the well-accepted optimum pH of 4-6 for *Komagataeibacter* to ‘produce cellulose’ was inaccurate. Instead, this pH range was a suitable condition for the gluconic oxidase.

According to the carbon metabolism pathway given by P. Ross et al., glycerol is the carbon source in the peripheral circuit of the glucose metabolism pattern of *Acetobacter xylinum* bacteria [47]. Hence, using glycerol as an alternative substrate may reduce the accumulation of gluconic acid and stabilize the pH. A previous report showed similarly that the BC yield of *Komagataeibacter rhaeticus* PG2 using glycerol as the carbon source was about 70% higher than that of glucose, and the pH remained stable [48]. Here, the result showed when using glycerol as the carbon source, the BC yield (4.93 g/l) was significantly higher than that of glucose (4.63 g/l) but lower than that of glucose/gluconic acid (5.58 g/l). However, the carbon conversion rate of glycerol to cellulose (25%) was higher than the media using glucose and/or gluconic acid as the source (23%), which indicates glycerol was a better carbon source for BC production. Besides, the pH of the glycerol medium only slightly decreased from 5 to 4.8, which indicates a limited production of the acidic substance. On the contrary, the glucose/gluconic acid carbon metabolism pathways involved more intermediate products, resulting in a decrease in pH (the pH of the media dropped from 5 to 4) and a comparatively lower conversion rate of the carbon source (Fig. 1D). Considering the cost and stability of the culture medium, glycerol was a more suitable carbon source for strain *K. hansenii* ATCC 53582.

### Crystalline Structure of BC Synthesized from Different Carbon Sources

**Fig. 2:**
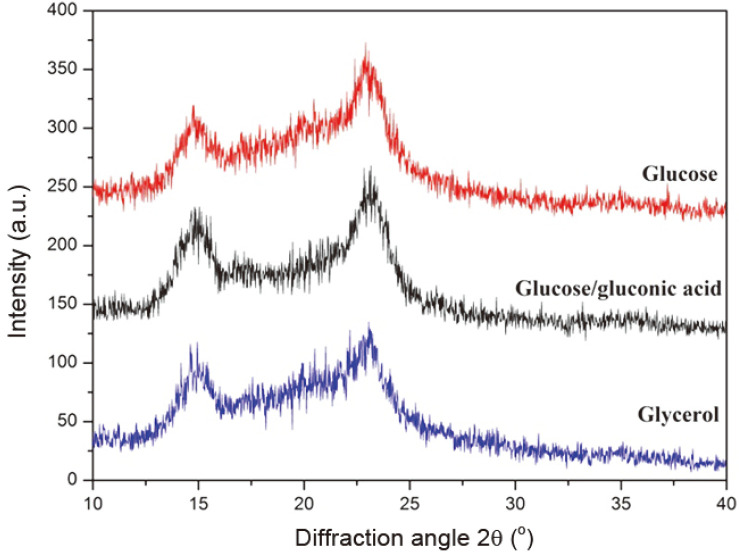
XRD diffraction patterns of BC produced from different carbon sources.

The XRD diffraction patterns of BC achieved from different carbon sources were shown in Fig. 2. All the diffraction patterns showed two sharp, strong peaks at 14.4° and 22.6° of 2θ, and a low intensity peak at 16.8° of 2θ, which represents a typical crystalline structure of cellulose I [49]. Additionally, the crystallinity and crystal sizes of BC produced in the different carbon sources had no significant differences (Table 3). Although the buffer (90 mM phosphate buffer) did not effectively control the drop of the pH when glucose was oxidized to gluconic acid, compared with glycerol as a carbon source, this weakly acidic environment had limilted influence on the crystalline structure.

### Ribbon Morphology of BC Synthesized from Different Carbon Sources

**Fig. 3:**
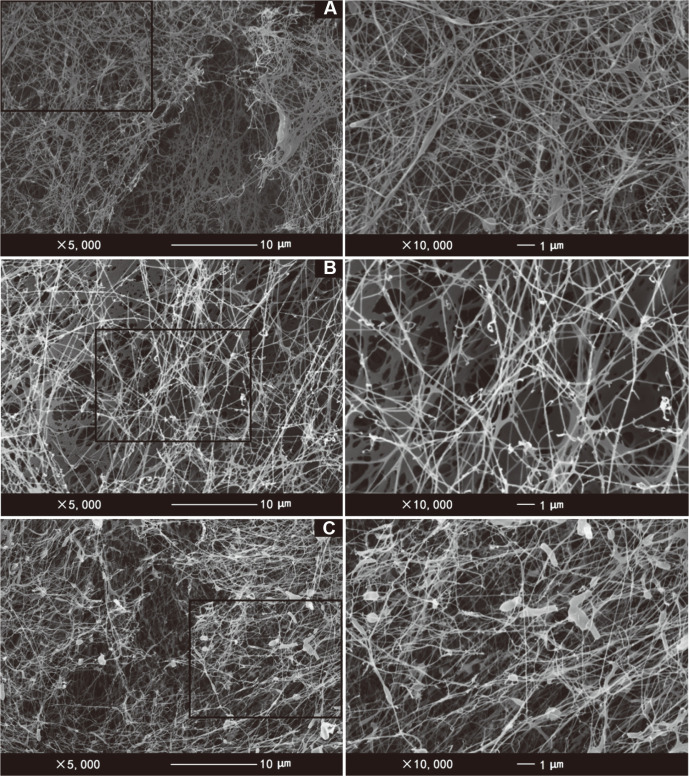
SEM images of freeze-dried BC: (A) glucose, (B) glycerol and (C) glucose/gluconic acid.

The BC produced from different carbon sources exhibited a typical fibril-network structure (Fig. 3). The average diameter of the cellulose ribbon achieved from the glucose/gluconic acid medium was the thinnest (47 ± 4.5 nm), whilst the BC obtained from HS medium and glycerol medium has a similar fiber diameter amounting to 61 ± 3.7 nm and 57 ± 6.1 nm, respectively. According to a previous report, the BC has no significant difference in terms of the fiber diameter when cultivated with the same bacterial strain in different carbon sources including glucose, mannitol, glycerol, fructose, sucrose and galactose [21]. However, a report showed increasing the concentration of water-soluble exopolysaccharide produced by *K. hansenii* ATCC 53582 can increase the diameter of cellulose ribbons [50]. Similarly, as one of the metabolites of *K. hansenii* ATCC 53582, increasing the concentration of gluconic acid may also affect the average size of BC ribbons.

### Mechanical Strength and Recovery Ability of BC Synthesized from Different Carbon Sources

**Fig. 4:**
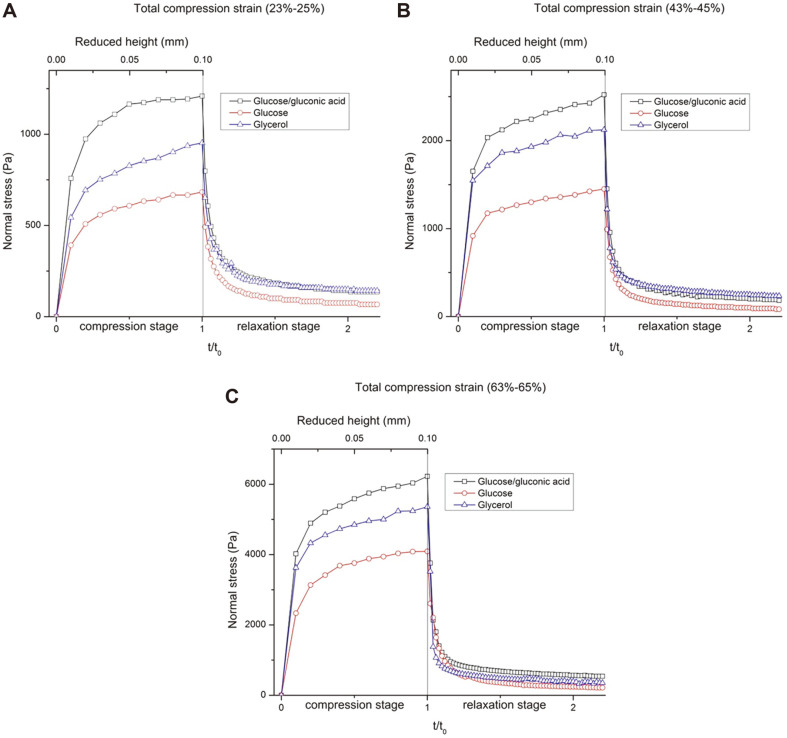
Normal stress of BC hydrogels produced from different carbons sources in the compressionrelaxation test.

The stiffness and recovery abilities of BC hydrogels produced from different carbon sources at low (23%-25%), medium (43%-45%) and high total compression strain (63%-65%) were compared (Fig. 4). Overall, in each cycle of compression-relaxation, all BC hydrogels exhibited a viscoelastic region (reduced height from 0 to 0.025 mm) and an apparently plastic deformation region (strain from 0.05 to 0.1 mm) under compression. The increasing rate in normal stress of BC produced from the glucose/gluconic acid carbon source was comparatively higher (Fig. 4). Generally, the external compressive force was mainly applied on the ribbons and their related junctions [51]. The concentration of the cellulose in the gel was a critical factor that affected the mechanical strength. Hence, the gel produced from the glucose/gluconic acid carbon source which had a higher concentration (1.8%) had a higher mechanical strength compared with the low-concentrated gels (1.3% and 1.1%, respectively) produced from glycerol and the sole glucose carbon source. The variation of the density was manifested in a decrease in the transparency of the hydrogel (materials, Fig. S3). The increasing rate in normal stress of all BC hydrogels at medium and high compression strain was about 2 and 4 times that at low compression strain (Fig. 4), respectively. The normal stress rate increased exponentially with the uniform increase of compression strain due to having a large number of fiber entanglements under high compression strain [16]. In addition, the fiber entanglement also inhibited the return of the water to the porous structure completely during relaxation stage, which greatly contributes to the normal stress [38]. When the applied normal stress was removed, all hydrogels exhibited time-dependent recovery behavior, in which the normal stress decreased in a short time (10 sec) and then recovered slowly. Due to the variation of the cellulose concentration, the gels achieved from glucose/gluconic acid and glycerol medium showed relatively lower recovery ability (recovery rate was 90% and 89% respectively) than that from glucose meidum (recovery rate was 93%), which may be due to the high entanglement level of the ribbons during the compression. This variation became narrow in the highly compressed samples as most of the ribbons had collapsed (Fig. 4C).

### Viscoelasticity of BC Synthesized from Different Carbon Sources

**Fig. 5:**
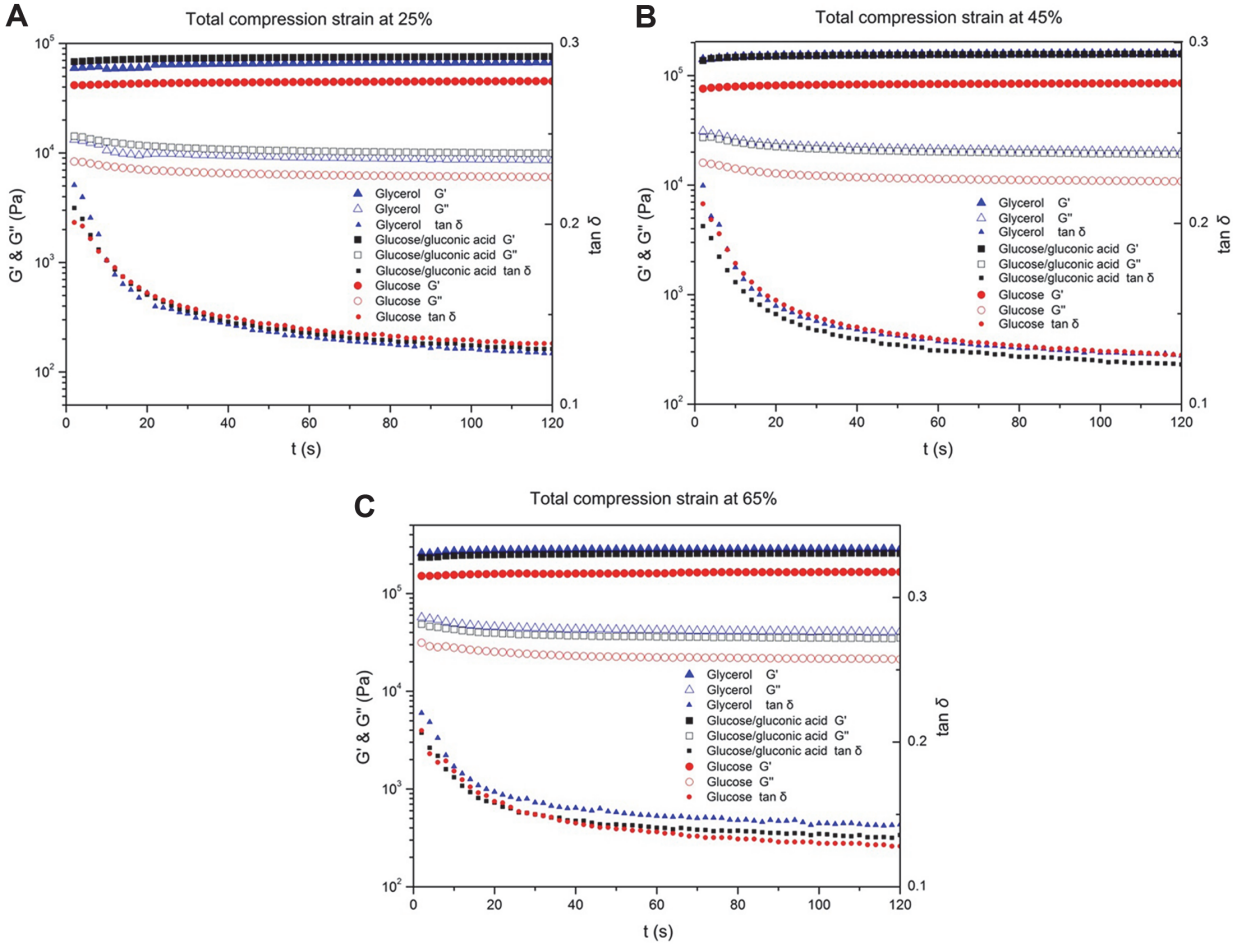
The storage modulus (G′), loss modulus (G′′) and loss factor (tan δ) of BC hydrogels.

The G′ value of all tested BC hydrogels was higher than the G′′ value, indicating that all of the BC hydrogels produced from different carbon sources exhibited a more pronounced elastic behavior than viscous behavior. The tan δ of all the samples dramatically dropped at the beginning stage of the oscillation (0-20 s), which was consistent with the result of the relaxation test. At this stage, when the normal compression was stopped, the water returned into the porous network and led to an increasing viscous behavior before reaching the equilibrium state. For the highly compressed samples, the decrease of tan δ was less significant due to the collapse of ribbons (Fig. 5C). The modulus of all samples was enhanced with the process of compression. The G′ of BC produced from glucose/gluconic acid and glycerol media remained higher than that from glucose media (Fig. 5). Generally, the G′ value depends on the number of fiber entanglements in the BC hydrogel, according to previous reports [52]. Hence, cellulose fibers in high-concentration BC gel increase the number of entanglements and lead to high G′ value [16]. According to a previous report [53], increasing the content of mannose-contained exopolysaccharide hydrolysate reduced the average distance between adjacent layers of BC, which makes the cellulose more compact and enhances the mechanical properties of the BC gel. This result indicates that the added gluconic acid and glycerol may be beneficial for the bacteria in building a strong gel than the single glucose carbon source.

## Conclusion

The starting pH and ionic strength of the buffer were both crucial for *K. hansenii* ATCC 53582 to synthesize BC when using glucose as the carbon source. The phosphate buffer (ionic strength = 90 mM) and phthalate buffer (ionic strength = 73 mM) were more effective than citrate buffer (ionic strength = 600 mM) and acetate buffer (ionic strength = 200-250 mM) for BC production. However, the accumulation of gluconic acid affected the pH of the cultural medium and cannot be effectively neutralized by the buffer. The gluconic acid was an intermediate compound for cellulose production. Adding gluconic acid (4 g/l) significantly enhanced the yield of BC but had limited influence on its crystalline structure. In addition, the bacterium used glycerol in a different carbon metabolism pathway which produced less acidic metabolites and maintained the pH stability. The BC produced from glucose/gluconic acid and glycerol carbon sources showed high mechanical strength and viscoelasticity, which were contributed by their high cellulose concentration. We believe this research will be beneficial in the future selection of production media to synthesize BC hydrogels with good texture properties.

## Supplemental Materials



Supplementary data for this paper are available on-line only at http://jmb.or.kr.

## Figures and Tables

**Table 1:** The different buffer solutions used in this research.

Buffer system	Starting pH	Constituents, concentration (N) and proportion (v/v)	
		0.2 N acetic acid	0.2 N sodium acetate
Acetate buffer solution	4.60	51%	49%
	5.00	30%	70%
	5.40	14%	86%
	5.80	6%	94%
		0.1 N citric acid	0.2 N disodium hydrogen phosphate
Phosphate buffer solution	4.60	53.25%	46.75%
	5.00	48.5%	51.5%
	5.40	44.25%	55.75%
	5.80	39.55%	60.45%
		0.1 N citric acid	0.1 N sodium citrate
Citrate buffer solution	4.60	44.5%	55.5%
	5.00	35%	65%
	5.40	25.5%	74.5%
	5.80	16%	84%
		0.1 N sodium hydroxide	0.1 N potassium hydrogen phthalate
^*^Phthalate buffer solution	4.60	11.1%	50%
	5.00	22.6%	50%
	5.40	34.1%	50%
	5.80	42.3%	50%

*Deionized water was used to fulfill the remaining proportion of phthalate.

**Table 2:** Effect of buffer and starting culture pH on BC yields.

Buffer	Ionic Strength (mM)	Actual starting pH	Final pH (9^th^ day)	Change of pH (9^th^ day)	BC yield (g/L)
Acetate	242	4.66	4.50*	-0.16	-
	230	5.05	4.95	-0.10	-
	214	5.47	5.34*	-0.13	-
	206	5.90	5.62**	-0.28	-
Phosphate	90	5.00	3.89**	-1.11	4.63±0.06^a^
	600	4.76	4.32**	-0.44	-
	600	5.03	4.45**	-0.58	-
	600	5.49	4.91**	-0.58	-
	600	5.86	5.26**	-0.60	-
Citrate	600	4.63	4.70*	0.07	-
	600	4.99	4.99	0	-
	600	5.29	5.47*	0.18	-
	600	5.67	5.75*	0.08	-
Phthalate	61	4.84	3.94**	-0.90	2.88±0.04^e^
	73	5.18	4.31**	-0.87	5.06±0.18^a^
	84	5.59	4.86**	-0.73	4.66±0.03^a^
	92	6.02	4.98**	-1.04	4.14±0.05^b,c^
Control group	－	4.68	3.16**	-1.52	3.98±0.08^c,d^
	－	5.08	3.28**	-1.80	4.27±0.07^b^
	－	5.46	3.32**	-2.14	4.03±0.09^c^
	－	5.83	3.35**	-2.48	3.75±0.12^d^

Change of pH= Final pH-Actual starting pH

(-) Indicates no obvious BC production

Significance analysis for actual starting pH and final pH; (*) and (**) denote statistically significant changes (LSD *t*-test, *p* value ≤ 0.05 and *p* value ≤ 0.01 respectively)

BC yield is presented as the mean ± SD for triplicate measurements. Means with different superscripts in the same column are considered statistically different (LSD *t*-test, *p* value ≤ 0.05)

**Table 3:** Crystallinity and crystal size of BC produced from different carbon sources.

^*^Carbon sources	CI (%)	D100 (nm)	D010 (nm)	D110 (nm)
Glucose	80.2±5.7^a^	3.2±0.4^a^	4.7±0.5^b^	3.1±0.4^a^
Glucose/gluconic acid	80.8±4.1^a^	3.7±0.3^a^	5.2±0.5^a,b^	3.4±0.2^a^
Glycerol	81.5±4.1^a^	3.4±0.3^a^	5.5±0.3^a^	3.3±0.2^a^

*Starting pH of the media for all carbon sources was 5.00.

All data were presented as the mean ± SD for triplicate measurements. Means with different superscripts in the same column were considered statistically different (LSD *t*-test, *p* value ≤ 0.05).
