# Supplementary material for: Effect of pH Buffer and Carbon Metabolism on the Yield and Mechanical Properties of Bacterial Cellulose Produced by Komagataeibacter hansenii ATCC 53582
Source: J Microbiol Biotechnol. 2020 Dec 16;31(3):429–38. doi: 10.4014/jmb.2010.10054 (PMC9705897; doi:10.4014/jmb.2010.10054)
Supplement: Supplementary file 1 [file jmb-31-3-429-supple.pdf]

https://doi.org/10.4014/jmb.2010.10054

**Supplementary materials**

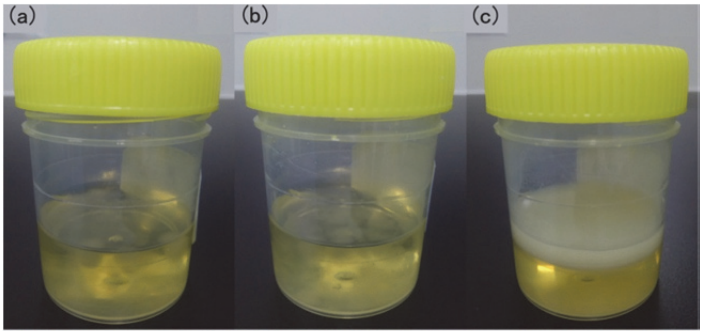


**Fig. S1. The synthesizing of BC in different buffer on 9th day of cultivation:** (a) 600 mM phosphate buffer, (b) 600 mM citrate buffer and (c) control group.

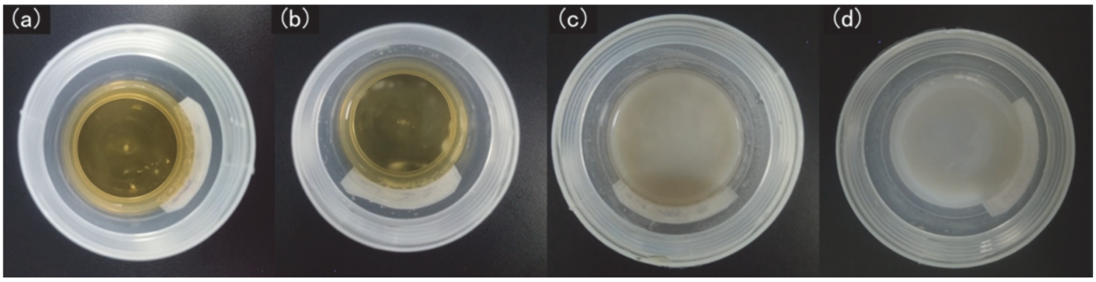


**Fig. S2. The synthesizing of BC in different buffer on 9th day of cultivation:** (a) 58 mM acetate buffer, (b) 115 mM acetate buffer, (c) 37 mM phthalate buffer and (d) 73 mM phthalate buffer.

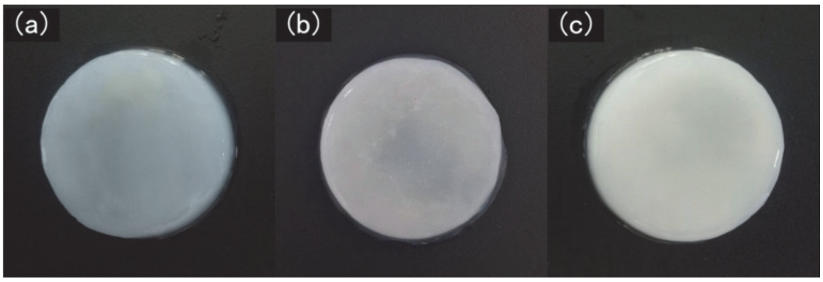


**Fig. S3. BC produced from different carbon sources:** (a) glucose, (b) glycerol and (c) glucose/gluconic acid.
